# lncRNA ADAMTS9-AS2/let-7a-5p axis regulates metabolic reprogramming by targeting HK2 in oral submucous fibrosis-associated oral squamous cell carcinoma

**DOI:** 10.1016/j.gendis.2025.101670

**Published:** 2025-05-05

**Authors:** Shanghui Zhou, Jingyu Zhan, Jia Wang, Jingang Yang, Dahe Zhang, Zhenming Li, Yue He

**Affiliations:** a Department of Oral and Maxillofacial-Head and Neck Oncology, Shanghai Ninth People’s Hospital, Shanghai Jiao Tong University School of Medicine, Shanghai 200011, China; b College of Stomatology, Shanghai Jiao Tong University, Shanghai 200011, China; c National Center for Stomatology, Shanghai 200011, China; d National Clinical Research Center for Oral Diseases, Shanghai 200011, China; e Shanghai Key Laboratory of Stomatology, Shanghai 200011, China; f Shanghai Research Institute of Stomatology, Shanghai 200011, China; g Shanghai Center of Head and Neck Oncology Clinical and Translational Science, Shanghai 200011, China; h Center of Stomatology, Hainan West Central Hospital, Hainan 570105, China; i Department of Preventive Dentistry, Shanghai Ninth People’s Hospital, Shanghai Jiao Tong University School of Medicine, Shanghai 200011, China; j Department of Endodontics, Shanghai Ninth People’s Hospital, Shanghai Jiao Tong University School of Medicine, Shanghai 200011, China; k Department of Stomatology, Tongren Hospital, Shanghai Jiao Tong University School of Medicine, Shanghai 200011, China; l Department of Oral Surgery, Shanghai Ninth People’s Hospital, Shanghai Jiao Tong University School of Medicine, Shanghai 200011, China; m Department of Oral & Cranio-Maxillofacial Surgery, Shanghai Ninth People’s Hospital, Shanghai Jiao Tong University School of Medicine, Shanghai 200011, China

**Keywords:** ADAMTS9-AS2, Aerobic glycolysis, HK2, let-7a-5p, OSCC, OSF

## Abstract

Oral squamous cell carcinoma in the background of/with oral submucous fibrosis (OSCC-OSF) has a unique etiology and is clinically distinct from other OSCCs. We previously identified ADAMTS9-AS2 as a functional tumor suppressor in OSCC-OSF through the regulation of PI3K-AKT signaling. However, its role in metabolic modulation and the underlying mechanisms remain unclear. In this study, we reported for the first time that ADAMTS9-AS2 suppressed aerobic glycolysis by cooperating with let-7a-5p in OSCC cells. Mechanistically, let-7a-5p inhibited HK2 expression by targeting its 3′-UTR, further deregulating glycolytic function, while enhancing HK2 expression rescued the inhibitory effects of the ADAMTS9-AS2/let-7a-5p axis on aerobic glycolysis and OSCC cell growth. Exosomal ADAMTS9-AS2 regulated metabolic reprogramming during OSCC tumorigenesis. ABC transporters in lipid and pyrimidine metabolism were significantly enriched pathways. Changes in several key metabolites were identified after ADAMTS9-AS2 exosome treatment, including increased levels of DL-glutamic acid and D-mannose, along with decreased levels of cytidine and D-maltose. Thus, our findings demonstrate that ADAMTS9-AS2 drives let-7a-5p binding to HK2 to suppress cell growth in OSCC by abolishing aerobic glycolysis. Our data on metabolic reprogramming have greatly expanded the role of the ADAMTS9-AS2/let-7a-5p axis as a key regulator of metabolism during OSCC tumorigenesis.

## Introduction

Oral squamous cell carcinoma (OSCC) is one of the most common malignant tumors of the head and neck, accounting for 90 % of its pathological types.^1^ Since its first description by Paymaster, oral submucous fibrosis (OSF) has emerged as a significant precancerous condition, with malignant transformation rates ranging from 3% to19%.^2^ Due to the specific pathogenesis associated with betel nut chewing, OSCC-OSF (oral squamous cell carcinoma in the background of/with oral submucous fibrosis) clinically manifests as a different disease from other OSCC.^3^, ^4^, ^5^ In recent years, betel nut chewing has spread throughout China mainland. Therefore, it is important to elucidate the key molecular events of OSF tumorigenesis for the prevention and early diagnosis of OSCC.

Metabolic dysfunction is one of the hallmarks of cancer, with deregulated cellular energy and glycolytic processes.^6^ Multiple mechanisms, such as genetic mutations, epigenetic changes, protein interactions, and enzyme–metabolite interactions influence the metabolic reprogramming of tumor cells.^7^ Studies have shown that increased glycolytic metabolism of OSCC cells can affect the metabolic reprogramming of OSF and activate them to become tumor-associated fibroblasts.^8^ ^1^H nuclear magnetic resonance spectroscopy also showed that the metabolites in the OSF group were significantly altered compared with the normal oral mucosa group.^9^ Thus, metabolic reprogramming may play an important role in OSF carcinogenesis, but the relevant molecular mechanisms remain unclear.

Long non-coding RNAs (lncRNAs) are aberrantly expressed in a variety of diseases and act as a scaffold for the interaction of miRNAs, mRNAs, and proteins. lncRNAs serve as oncogenes or tumor suppressor genes to participate in tumor initiation and progression.^10^ We have previously interpreted the lncRNA expression profiles at different stages of OSF carcinogenesis.^11^ We found that ADAMTS9-AS2 was significantly down-regulated in OSCC-OSF, correlated with poor survival of OSCC patients.^12^ Exosomal ADAMTS9-AS2 inhibits the AKT signaling pathway and regulates epithelial–mesenchymal transition, thereby suppressing OSCC cell growth, migration, and invasion.^12^ By profiling the miRNA expression profile of exosomal ADAMTS9-AS2, the metabolic pathway was the most significantly enriched, indicating metabolic deregulation in the malignant progression of OSCC by interacting with miRNAs.^12^ However, the underlying mechanisms by which exosomal ADAMTS9-AS2 regulates metabolism in OSCC are largely unknown.

In this study, we investigated the role of ADAMTS9-AS2 in reprogramming cancer metabolism in OSCC. We showed that ADAMTS9-AS2 and let-7a-5p suppressed aerobic glycolysis in OSCC cells. We discovered that the ADAMTS9-AS2/let-7a-5p axis regulated the metabolism of OSCC cells by targeting hexokinase 2 (HK2). Importantly, we further demonstrated that HK2 expression was critical for ADAMTS9-AS2-mediated cell proliferation. Thus, our findings provide a novel mechanism by which the ADAMTS9-AS2/let-7a-5p axis inhibits the Warburg effect and OSCC progression.

## Materials and methods

### Cell lines, tumor samples, and normal tissues

OSCC cell lines (CAL27 and SCC9) and normal human oral keratinocytes (HOK) used in this study were obtained from the American Type Culture Collection. Cells were cultured in complete Dulbecco’s modified Eagle medium supplemented with 10% fetal bovine serum (Gibco-BRL) or in its combination with Ham’s F-12 nutrient mixture medium (Gibco-BRL) supplemented with 10% fetal bovine serum. The normal human oral keratinocyte cell line was cultured in a dermal cell basal medium plus a keratinocyte growth kit.

DNA obtained from OSCC, OSF, and normal oral mucosa tissues has been described previously.^11^^,^^12^ This study was approved by the Institutional Review Board of Shanghai Jiaotong University School of Medicine.

### Quantitative reverse transcription PCR

Total RNA was extracted from harvested cells or tissues using Trizol reagent (Invitrogen, Carlsbad, California, USA). RNA was then reverse-transcribed into cDNAs using the ExScript RT-PCR kit (TaKaRa, Japan). For real-time PCR analysis, the primers for *ADAMTS9-AS2* were 5′- GAAGGATGTGCTTGGGAACT-3′ (forward) and 5′-CATGGTAAAGGCTGGTCAGA-3′ (reverse). The control primers (*ACTB*) were 5′- GCGTGACATTAAGGAGAAGC-3′ (forward) and 5′-CCACGTCACACTTCATGATGG-3′ (reverse). The primers for *let-7a-5p* were 5′-CGGTGAGGTAGTAGGTTG-3′ (forward) and 5′-GCAGGGTCCGAGGTATTC-3′ (reverse). The control primers (*U6*) were 5′-CTCGCTTCGGCAGCACA-3′ (forward) and 5′-AACGCTTCACGAATTTGCGT-3′ (reverse). Quantitative reverse transcription PCR was performed using a SYBR Green PCR Master Mix Kit (Invitrogen) on the ABI StepOne Real-Time PCR System (Applied Biosystems). The relative expression of genes studied was estimated using the threshold cycle (Ct) method, and all assays were performed in triplicate.

### siRNA transfection

siRNA oligos targeting ADAMTS9-AS2 were purchased from GenePharma (Suzhou, China). siRNAs targeting ADAMTS9-AS2 are as follows: si-AS2-1-sense: 5′- CAGAGACGCAGGUAUUUAUTT-3′; si-AS2-1-antisense: 5′- AUAAAUACCUGCGUCUCUGTT-3′; si-AS2-2-sense: 5′- CGGCUUUCAAGAUUGGAAUTT-3′; si-AS2-2-antisense, 5′- AUUCCAAUCUUGAAAGCCGTT-3′. siRNAs targeting HK2 (SR302109) were purchased from Origene (Origene, Rockville, Maryland, USA). siRNAs were transfected into CAL27 and SCC9 cell lines using Lipofectamine 3000 (Invitrogen, USA) according to the manufacturer’s instructions.

### Let-7a-5p mimics and antagomirs

Let-7a-5p mimics and inhibitors were purchased from GenePharma (Suzhou, China). An amount of 50 nM let-7a-5p mimics or 100 nM antagomirs was transfected into CAL27 and SCC9 cells using Lipofectamine RNAiMAX (Invitrogen, USA) according to the manufacturer’s instructions. The let-7a-5p inhibitor sequence is AACUAUACAACCUACUACCUCA.

### Western blot analysis

Cells were lysed in RIPA lysis buffer containing protease inhibitors as previously described.^12^ Equal amounts of protein were separated by SDS-PAGE and transferred to nitrocellulose membranes, followed by incubation with the indicated antibodies at 4 °C overnight and detection by enhanced chemiluminescence (Promega, USA). Antibodies used in this study are as follows: anti-HK2 (Cell Signaling Technology, #2867), anti-PKM (Santa Cruz, sc-365684), anti-LDHA (Cell Signaling Technology, #3582), and anti-GAPDH (Santa Cruz, sc-32233).

### Luciferase reporter assay

Wild-type and mutated putative binding sites of let-7a-5p on the HK2 3′-UTR based on the prediction database were cloned into the pmirGLO dual-luciferase miRNA target expression vector (Promega, USA). For reporter activity analysis, cells were transfected with the wild-type or mutated HK2 3′-UTR reporter and let-7a-5p mimics or negative control using Lipofectamine 3000. Luciferase reporter assays and luciferase activity detection were performed according to the manufacturer’s instructions (Promega, USA).

### Lactate production

The Lactate-Glo™ assay kit (Promega, USA) was used to measure extracellular lactate production according to the manufacturer’s protocols. Cells were seeded in 96-well plates at 10,000 cells per well, incubated in a culture medium for 48 h, and then collected for further luminescence detection to assess lactate production.

### Glucose uptake

The Glucose Uptake-Glo™ Assay Kit (Promega, USA) was used to measure intracellular glucose according to the manufacturer’s protocols. Cells were seeded in 96-well plates for culture and then washed with phosphate buffer saline after the medium was removed. 50 μL of the prepared 1 mM 2DG was added per well and incubated at room temperature for 10 min, followed by the addition of 100 μL of 2DG6P detection reagent. Luminescence was detected to assess glucose uptake.

### ATP production

ATP levels were measured using the CellTiter-Glo® 2.0 Cell Viability Assay (Promega, USA) according to the manufacturer’s protocols. Luminescence was measured to assess ATP production.

### Oxygen consumption rate

The Extracellular Oxygen Consumption Rate Plate Assay Kit (Dojindo China Co., Ltd.) was used to measure the oxygen consumption rate according to the manufacturer’s protocols. Cells were suspended in the working solution containing the oxygen probe, and the microplate was incubated for 30 min in a microplate reader (37 °C). After the addition of the working solution, 1 drop of mineral oil was added to the cells for stimulation. The kinetics were measured using the microplate reader, and the oxygen consumption rate was further calculated from the intensity.

### CCK-8 assay

Cell proliferation assay was performed using Cell Counting Kit 8 Assay Kit (Dojindo China Co., Ltd.) according to the manufacturer’s protocols. Cells were seeded in 96-well plates and cultured for the indicated hours. Amount of 10 μL of CCK-8 solution was added to each well and incubated for 4 h, and the absorbance value was detected at 450 nm. All experiments were repeated in triplicate.

### Liquid chromatography-mass spectrometry for untargeted metabolomic analysis

Untargeted metabolomics was performed by Genechem Co. (Shanghai, China) using ultra-high-performance liquid chromatography (UHPLC) & high-resolution mass spectrometry. Chromatographic separation was performed on an AB TripleTOF 6600 system (AB SCIEX) using a 1290 Infinity II LC system (Agilent, Santa Clara). Twelve exome samples from the control and ADAMTS9-AS2 expressing groups were prepared for liquid chromatography-mass spectrometry analysis, followed by qualitative and quantitative metabolite analysis, data quality assessment, and data analysis.

### Data processing

Raw data in Wiff format were converted to mzXML format by ProteoWizard, and then XCMS software was used for peak alignment, retention time correction, and peak area extraction. For the data extracted by XCMS, metabolite structure identification and data pre-processing were performed first, followed by experimental data quality assessment and final data analysis. Data analysis content included univariate statistical analysis, multidimensional statistical analysis, differential metabolite screening, differential metabolite correlation analysis, and Kyoto Encyclopedia of Genes and Genomes (KEGG) pathway analysis.

### Statistical analysis

Data were presented as mean ± standard deviation. Student’s *t*-tests were used to compare the means of the two groups. One-way ANOVA was subjected to comparisons between groups. Statistical analyses were performed with GraphPad Prism 8 software. *P* values < 0.05 were considered statistically significant. All experiments were performed in triplicate and repeated three times.

## Results

### Exosome-derived ADAMTS9-AS2 suppresses the Warburg effect in OSCC cells

**Figure 1:**
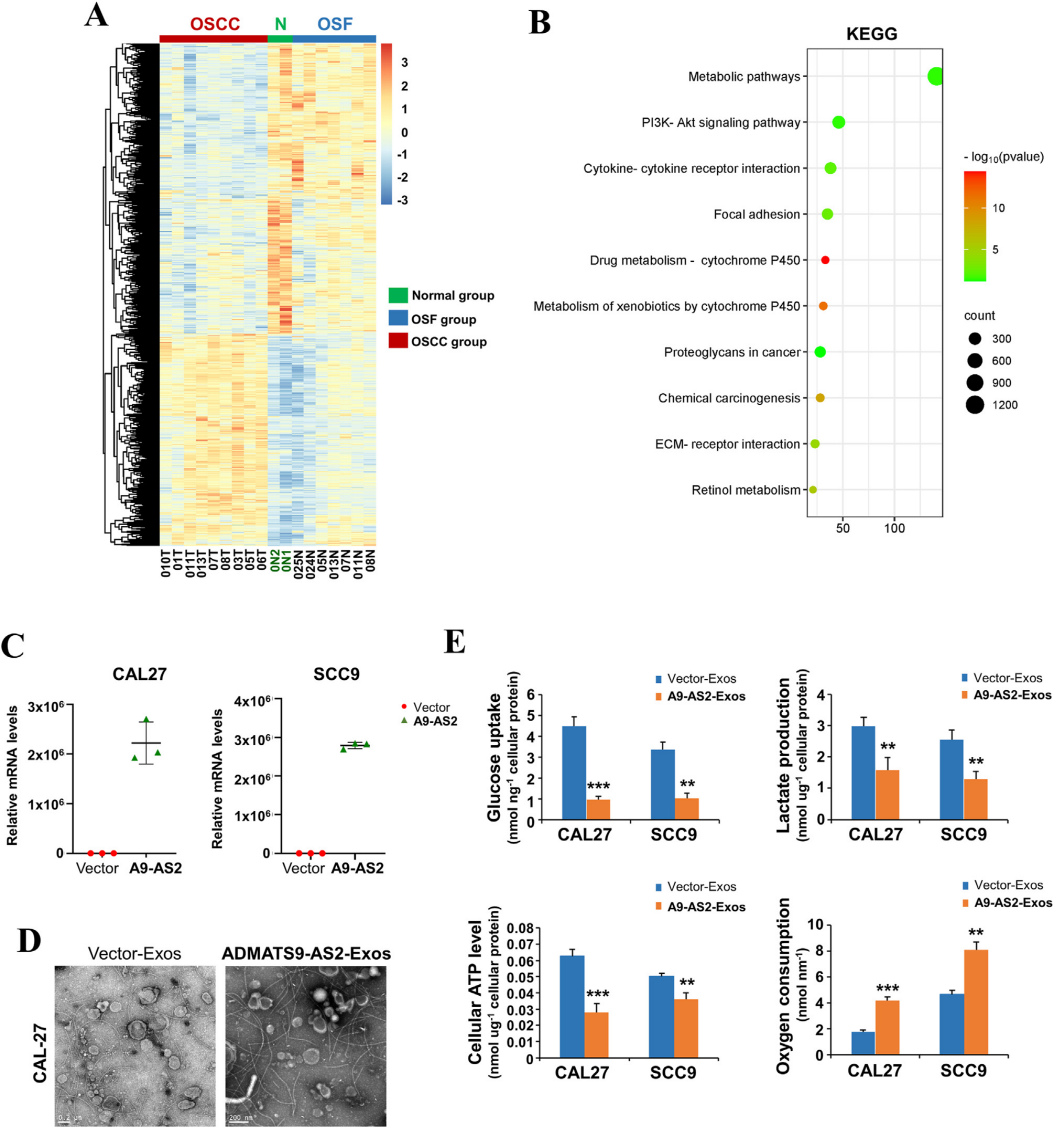
ADAMTS9-AS2 suppresses the Warburg effect in OSCC cells. **(A)** The heatmap showing the clustering of samples from normal oral mucous, OSF, and OSCC-OSF tissues based on gene and transcript expression. **(B)** The top 10 most significantly enriched KEGG pathways among differentially expressed genes, ranked by enrichment score (−log_10_ (*P*-value)). **(C)** Quantitative reverse transcription PCR analysis of ectopic ADAMTS9-AS2 expression was conducted on CAL27 and SCC9 OSCC cell lines. *ATCB* was used as control RNAs. **(D)** Exosomes isolated from supernatants of ADAMTS9-AS2-expressing OSCC cells were analyzed by transmission electron microscopy. Scale bar = 200 nm. **(E)** Glucose uptake, lactate production, cellular ATP levels, and oxygen consumption were measured in CAL27 and SCC9 cells treated with ADAMTS9-AS2 or vector-expressing exosomes. The data were presented as mean ± standard deviation of three independent experiments by student’s *t*-test. ∗∗*P* < 0.01, ∗∗∗*P* < 0.001. A9-AS2, ADAMTS9-AS2; Exos, exosomes; OSCC, oral squamous cell carcinoma; OSF, oral submucous fibrosis.

To identify key transcripts during OSF malignant transformation, we extracted our previous RNA sequencing data in 2 normal oral mucous, 7 OSF tissues, and 9 OSCC-OSF tissues (Fig. 1A; Gene Expression Omnibus ID GSE106534). KEGG pathway analysis of differentially expressed genes during OSF malignant transformation showed that the metabolism pathway was the most significantly enriched pathway (Fig. 1B).

We previously identified ADAMTS9-AS2 down-regulation in OSCC tissues compared with normal oral mucous and OSF tissues.^12^ OSCC cells (CAL27, SCC9) that stably express ADAMTS9-AS2 have been established using virus infection followed by puromycin selection.^12^ The exosomes were also isolated and identified from the culture medium of an ADAMTS9-AS2 or vector-expressing OSCC cell line (CAL27) by transmission electron microscopy and found to have a typical circular morphology (Fig. 1D).

We next tested a potential metabolic deregulation by exosome-derived ADAMTS9-AS2 in OSCC cells. Measurement of metabolic parameters revealed that cellular glucose uptake and lactate production in the culture medium were significantly reduced in ADAMTS9-AS2 exosome-treated CAL27 and SCC9 cells, accompanied by increased cellular oxygen consumption rates (Fig. 1E). These results suggest that exosome-derived ADAMTS9-AS2 suppresses the Warburg effect in OSCC cells.

### Accumulation of let-7a-5p upon ADAMTS9-AS2 expression suppresses the Warburg effect in OSCC cells

**Figure 2:**
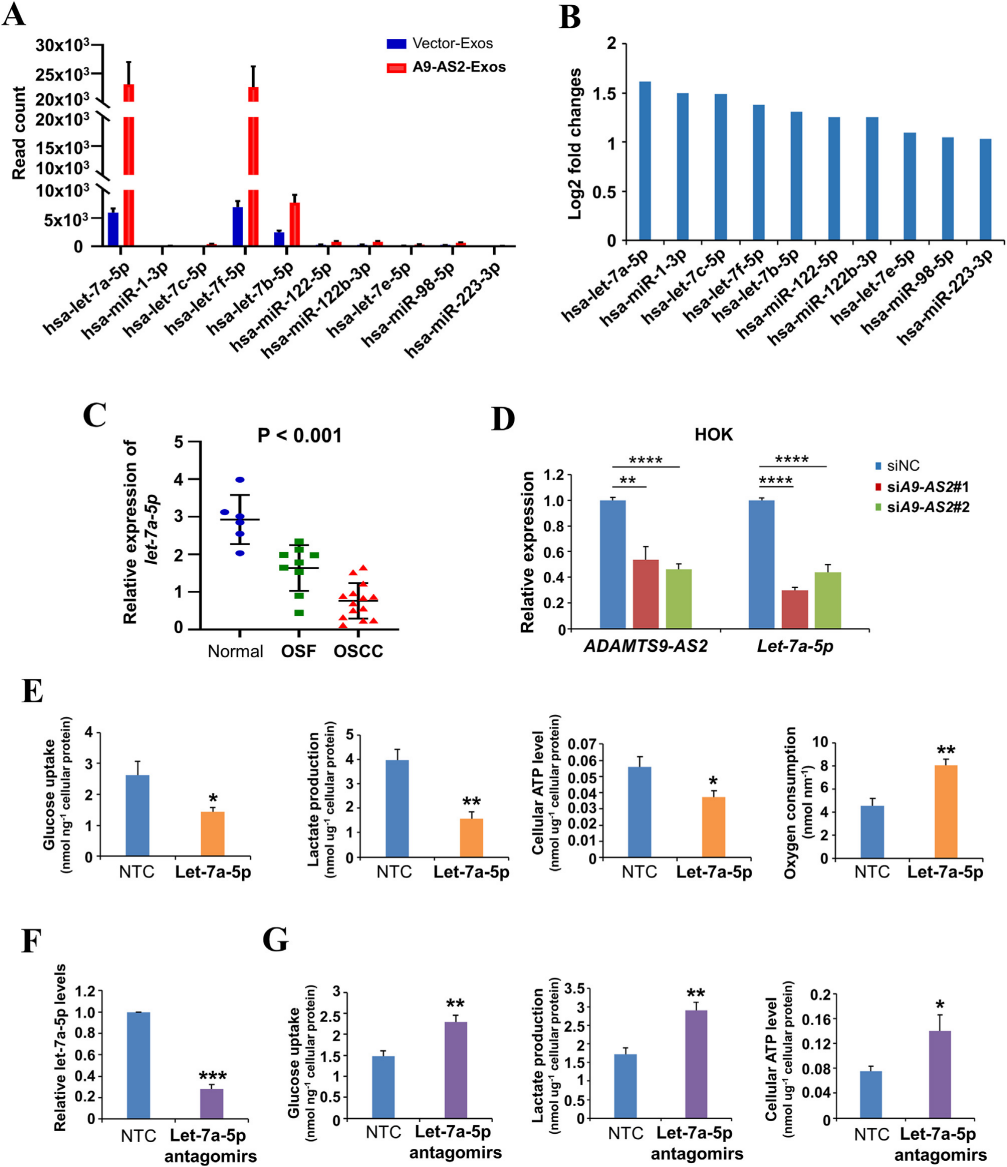
Let-7a-5p suppresses the Warburg effect in oral squamous cell carcinoma cells. **(A, B)** Expression levels (A) and fold changes (B) of the top 10 differential miRNA expression by exosomal ADAMTS9-AS2 in CAL27 cells. **(C)** Relative let-7a-5p expression in samples of patients at different stages of the disease. **(D)** Quantitative reverse transcription PCR was used to analyze ADAMTS9-AS2 and let-7a-5p levels after ADAMTS9-AS2 knockdown in HOK cells. **(E)** Glucose uptake, lactate production, ATP levels, and O_2_ consumption were measured in CAL27 cells transfected with non-targeting control (NTC) or let-7a-5p mimics. **(F)** Quantitative reverse transcription PCR analysis of let-7a-5p expression in CAL27 cells transfected with NTC or let-7a-5p antagomirs. Glucose uptake, lactate production, and ATP levels were measured in CAL27 cells transfected with NTC or let-7a-5p antagomirs. The data were presented as mean ± standard deviation of three independent experiments by student’s *t*-test. ∗*P* < 0.05, ∗∗*P* < 0.01, ∗∗∗*P* < 0.001. A9-AS2, ADAMTS9-AS2; Exos, exosomes.

Our previous work showed that exosomal ADAMTS9-AS2 functioned by interacting with miRNAs during OSF progression.^12^ Through analyzing the miRNA expression profile, we observed that mature let-7a-5p was the most significantly up-regulated miRNA regulated by exosomal ADAMTS9-AS2 in CAL27 cells (Fig. 2A), and the log_2_ fold change is about ∼1.6 with mostly significant adjusted *P* value (*P*_adj_ = 4.9685E-149) (Fig. 2B). We next analyzed expression levels of let-7a-5p in normal mucous, OSF, and OSCC samples by quantitative reverse transcription PCR analysis and observed that let-7a-5p expression was down-regulated in OSCC tissues compared with OSF and normal mucous tissues (^∗∗∗^*P* < 0.01) (Fig. 2C). We further investigated the potential relationship between ADAMTS9-AS2 and let-7a-5p by knocking down ADAMTS9-AS2 by siRNA in HOK cells with endogenously expressed ADAMTS9-AS2. We found that ADAMTS9-AS2 inhibition significantly decreased let-7a-5p expression compared with the control group (Fig. 2D). These results suggest that ADAMTS9-AS2 may exert its biological functions by interacting with let-7a-5p in OSCC cells.

To determine whether let-7a-5p regulates the metabolism of OSCC cells, we transfected CAL27 and SCC9 cells with miRNA mimics of let-7a-5p. Consistent with the effect of ADAMTS9-AS2, forced expression of let-7a-5p significantly reduced glucose uptake, lactate production, and ATP levels, while significantly increasing O_2_ consumption compared with the non-targeting control group (Fig. 2E). Furthermore, inhibition of let-7a-5p by antagomirs resulted in enhanced glucose uptake, lactate production, and ATP levels in CAL27 and SCC9 cells (Fig. 2F, G). These results suggest that let-7a-5p is involved in the suppression of aerobic glycolysis, or the Warburg effect, in OSCC cells.

### ADAMTS9-AS2 regulates aerobic glycolysis via HK2 in OSCC cells

**Figure 3:**
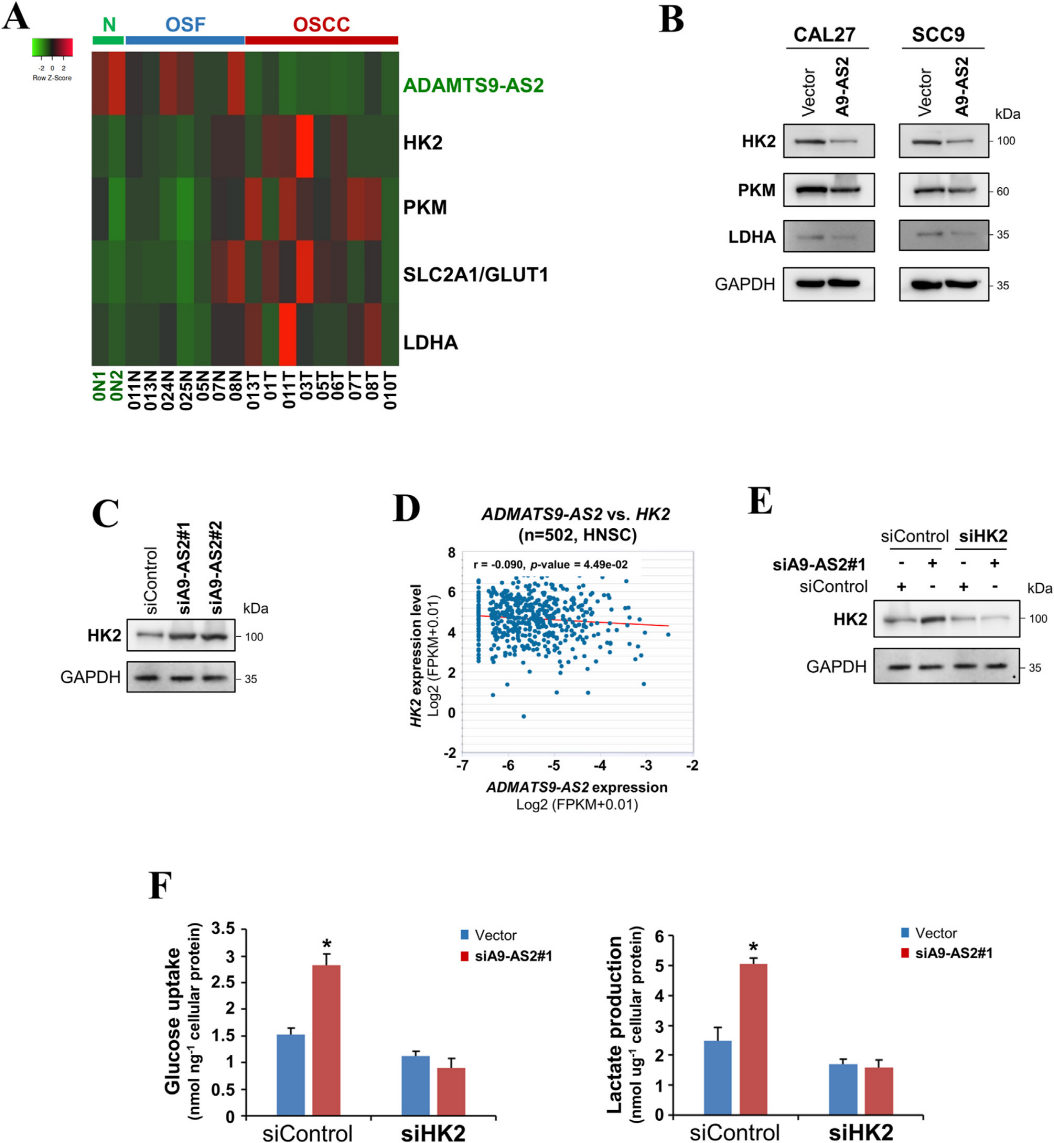
ADAMTS9-AS2 regulates aerobic glycolysis via HK2. **(A)** The heatmap showing aerobic glycolysis-related genes differentially expressed among normal oral mucous, OSF, and OSCC-OSF samples. **(B)** Protein levels of metabolic enzymes were determined by western blot in CAL27 and SCC9 cells expressing ADAMTS9-AS2 and empty vector. GAPDH was used as a loading control. **(C)** HK2 protein levels were determined by western blot in HOK cells with ADAMTS9-AS2 knockdown. **(D)** Correlation between *ADAMTS9-AS2* and *HK2* analyzed using starBase/ENCORI database. **(E)** HK2 protein expression level in HOK cells with ADAMTS9-AS2 and/or HK2 knockdown. GAPDH was used as a loading control. **(F)** Cellular glucose uptake and lactate production in the medium of HOK cells with ADAMTS9-AS2 and/or HK2 knockdown were determined. The data were presented as means ± standard deviation of three independent experiments by student’s *t*-test. ∗*P* < 0.05. A9-AS2, ADAMTS9-AS2; OSCC, oral squamous cell carcinoma; OSF, oral submucous fibrosis.

Given that ADAMTS9-AS2 promoted let-7a-5p expression in OSCC cells, we investigated whether ADAMTS9-AS2 suppressed the Warburg effect by up-regulating let-7a-5p target genes associated with glycolysis. We first evaluated the mRNA expression of key enzymes involved in glucose metabolism in normal mucosa, OSF, and OSCC tissues, and observed that OSCC tissues with low expression of ADAMTS9-AS2 had higher levels of glucose metabolism-related genes (HK2, PKM, SLC2A1/GLUT1, and LDHA) compared with normal mucosa and OSF tissues (Fig. 3A). Western blot analysis further showed that overexpression of ADAMTS9-AS2 resulted in a significant decrease in HK2 and a mild down-regulation of pyruvate kinase M (PKM) and lactate dehydrogenase A (LDHA) protein expression in CAL27 and SCC9 cells (Fig. 3B). Consistently, down-regulation of ADAMTS9-AS2 by siRNA reduced HK2 protein levels in HOK cells (Fig. 3C). Using the starBase/ENCORI database, we also explored the potential correlation between *ADAMTS9-AS2* and *HK2* in head and neck squamous cell carcinoma (HNSCC) dataset and found an inverse correlation between *ADAMTS9-AS2* and *HK2* in HNSCC tumor samples (Fig. 3D).

As HK2 is a critical enzyme in the regulation of glycolysis in cancer cells, we next investigated whether HK2 was involved in the inhibition of ADAMTS9-AS2-regulated glycolysis in OSCC cells. Results showed that when HK2 was knocked down by siRNAs in HOK cells, the increase in glucose uptake and lactate production induced by ADAMTS9-AS2 knockdown was attenuated (Fig. 3E, F), suggesting that HK2 is a functional downstream target of ADAMTS9-AS2 to regulate aerobic glycolysis.

### Let-7a-5p inhibits HK2 expression by directly targeting its 3′-UTR

**Figure 4:**
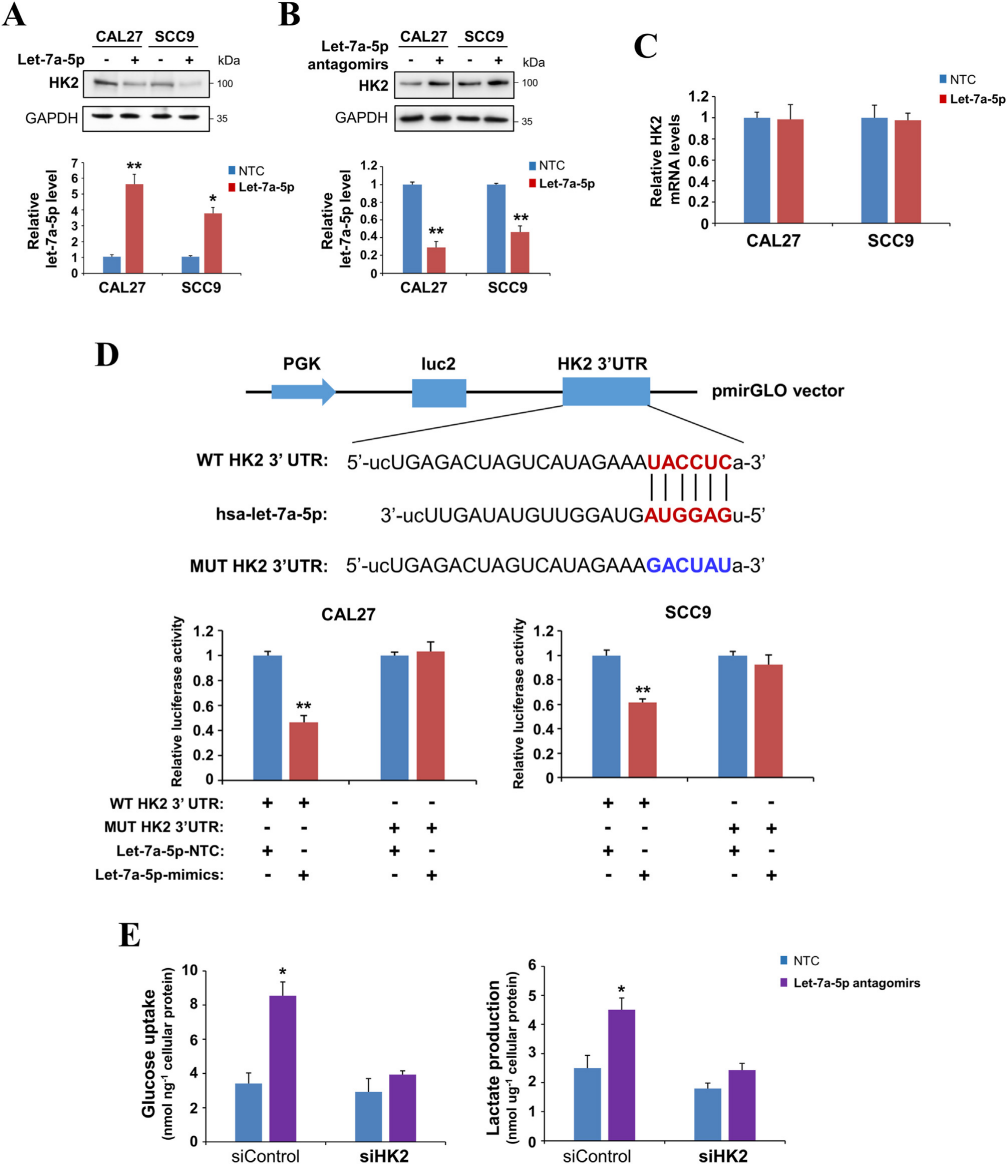
Let-7a-5p inhibits HK2 expression by directly targeting its 3′-UTR in oral squamous cell carcinoma cells. **(A, B)** Immunoblot analysis of HK2 expression in CAL27 and SCC9 cells transfected with non-targeting control (NTC), (A) let-7a-5p mimics, or (B) let-7a-5p antagomirs. Histograms show relative let-7a-5p expression by quantitative reverse transcription PCR. **(C)** HK2 mRNA levels were measured by quantitative reverse transcription PCR in CAL27 and SCC9 cells expressing NTC or let-7a-5p mimics. **(D)** The top panel indicates wild-type (WT) and mutated (Mut) forms of putative let-7a-5p target sequences of HK2 3′-UTR. The red font indicates the putative let-7a-5p binding sites within human HK2 3′-UTR. The blue font indicates the mutations introduced into the HK2 3′-UTR. Let-7a-5p mimics were co-transfected with pmirGLO-HK2-3′-UTR or pmirGLO-HK2-3′-UTR-mut into CAL27 and SCC9 cells, followed by dual-luciferase analysis. **(E)** NTC or let-7a-5p antagomirs were transfected into CAL27 and SCC9 cells with HK2 knockdown, followed by measurement of cellular glucose uptake and lactate production in culture media. The data were presented as mean ± standard deviation of three independent experiments by student’s *t*-test. ∗*P* < 0.05, ∗∗*P* < 0.01.

We next analyzed the potential relationship between HK2 and let-7a-5p in OSCC cells by transfecting let-7a-5p mimics and antagomirs. We found that let-7a-5p mimics significantly suppressed HK2 protein expression in both CAL27 and SCC9 cells (Fig. 4A), whereas let-7a-5p antagomirs increased HK2 protein expression (Fig. 4B), suggesting that HK2 may be a target gene of let-7a-5p. Further experiments showed that let-7a-5p did not affect HK2 mRNA levels (Fig. 4C), indicating that HK2 is regulated by let-7a-5p at the post-transcriptional level.

Bioinformatic analysis revealed the presence of a potential let-7a-5p binding fragment in the 3′-UTR of the HK2 gene. We then cloned this potential let-7a-5p binding fragment and its mutant form into a luciferase-expressing pmirGLO reporter vector (Fig. 4D). We found that the expression of let-7a-5p significantly suppressed the activity of the luciferase reporter gene containing the wild-type 3′-UTR of HK2 but not that containing the mutant form (Fig. 4D), indicating that HK2 is a direct target of let-7a-5p. We next investigated whether HK2 was involved in the decreased glycolytic regulated by let-7a-5p in OSCC cells. We found that the effect of let-7a-5p antagomirs on glucose uptake and lactate production was abolished in HK2-knockdown CAL27 cells (Fig. 4E), establishing HK2 as a functional downstream target of let-7a-5p in the regulation of glycolytic metabolism.

### ADAMTS9-AS2/HK2 axis regulates cell proliferation via glycolysis in OSCC cells

**Figure 5:**
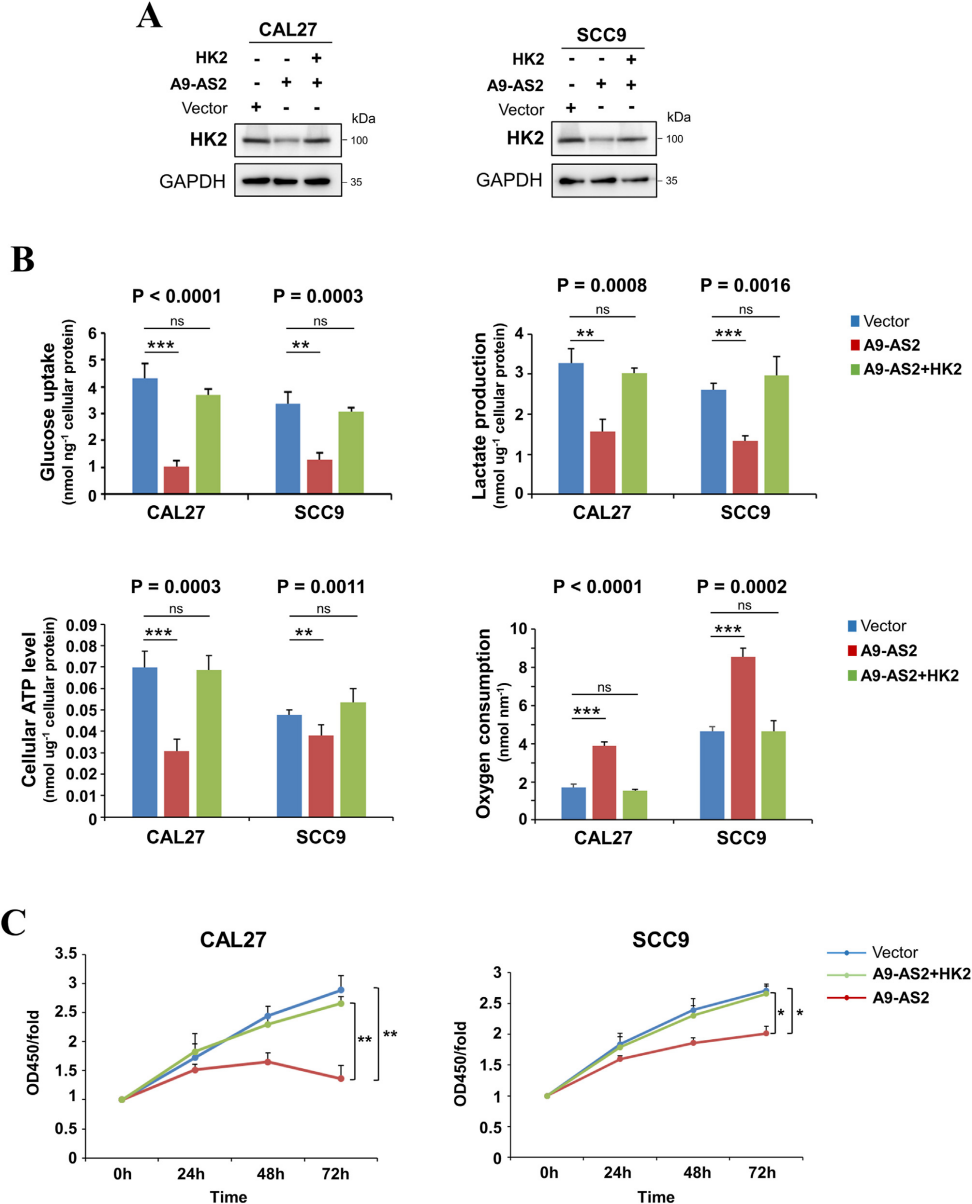
The ADAMTS9-AS2/let-7a-5p/HK2 axis regulates glycolysis in oral squamous cell carcinoma cells. **(A)** Representative immunoblot reveals the expression level of HK2 in ADAMTS9-AS2-expressing CAL27 and SCC9 cells transfected with HK2 expression or control plasmids. **(B)** Glucose uptake, lactate production, cellular ATP levels, and oxygen consumption were measured in ADAMTS9-AS2-expressing CAL27 and SCC9 cells transfected with HK2 expression plasmid, compared with controls. **(C)** Cell viability in CAL27 and SCC9 cells with both ADAMTS9-AS2 and HK2 expression was determined by CCK8 assay. The data were presented as mean ± standard deviation of three independent experiments by one-way ANOVA with multiple comparison post hoc analysis. ∗*P* < 0.05, ∗∗*P* < 0.01. A9-AS2, ADAMTS9-AS2.

We further investigated the effect of the ADAMTS9-AS2/HK2 axis on aerobic glycolysis in OSCC cells. Ectopic expression of ADAMTS9-AS2 decreased glucose uptake and lactate and ATP production in OSCC cells, which could be reversed by the re-expression of HK2 in the ADAMTS9-AS2-expressing CAL27 and SCC9 cells (Fig. 5A, B). We then examined the effect of ADAMTS9-AS2/HK2 axis on oxygen consumption. As expected, let-7a-5p mimics showed increased oxygen consumption rate in OSCC cells, and re-expression of HK2 in the ADAMTS9-AS2 expressing cells rescued these effects (Fig. 5A, B). These data suggest that the ADAMTS9-AS2/HK2 axis regulates aerobic glycolysis in OSCC cells.

Since ADAMTS9-AS2 suppressed OSCC cell proliferation, migration, and invasion, we explored whether the ADAMTS9-AS2/HK2 axis exerted its tumor-suppressive functions through glycolysis. Indeed, ADAMTS9-AS2-mediated inhibition of tumor cell proliferation was reversed by the re-expression of HK2 in CAL27 and SCC9 cells. These data suggest that the ADAMTS9-AS2/HK2 axis modulates OSCC cell proliferation mainly through glycolysis (Fig. 5C).

### Metabolic reprogramming in OSCC cells modulated by ADAMTS9-AS2-exosomes

**Figure 6:**
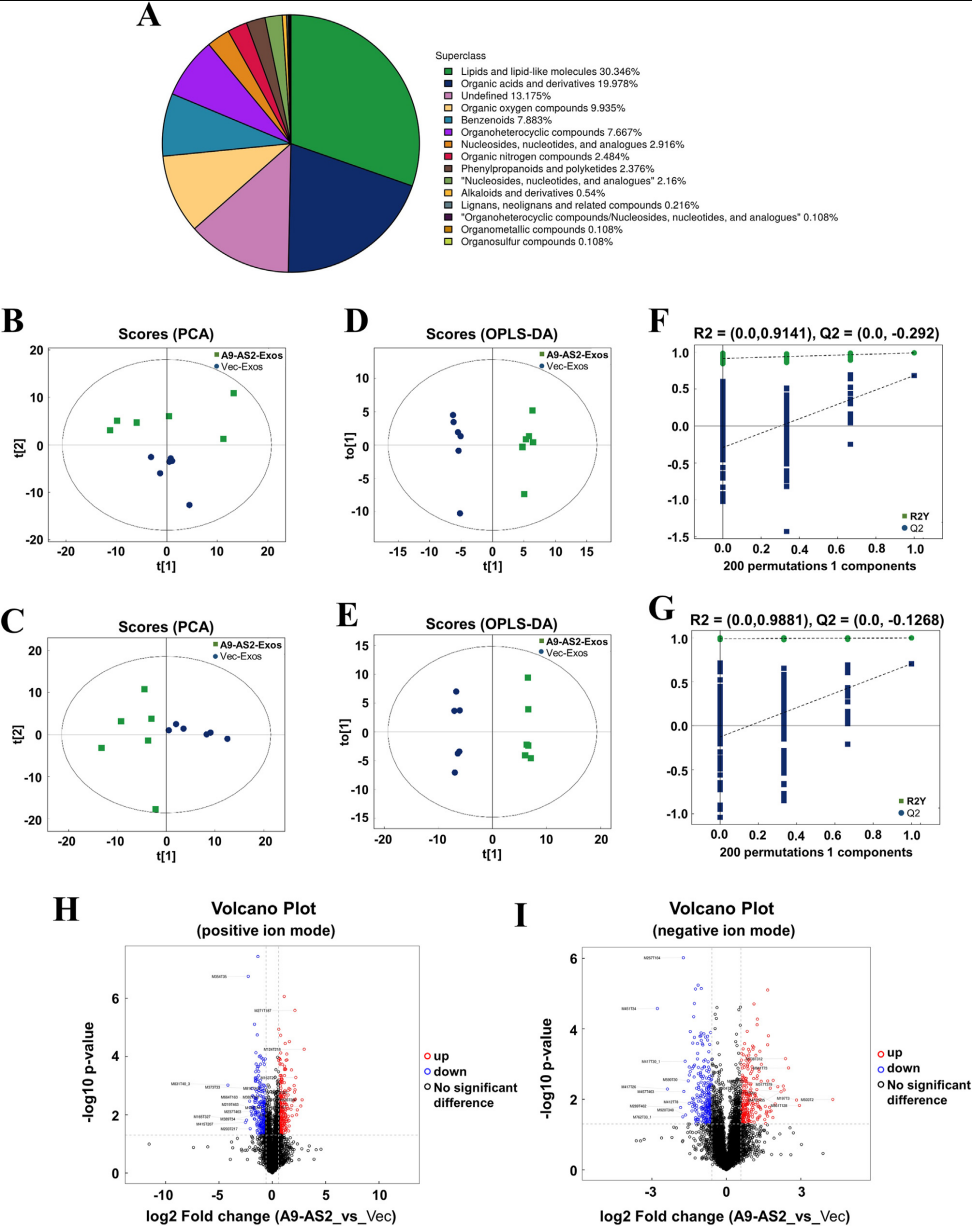
Liquid chromatography-mass spectrometry profiling of metabolomic reprogramming in CAL27 cells after ADAMTS9-AS2-Exos treatment and identification of metabolites. **(A)** The proportion of the identified metabolites in each chemical classification. Classification statistics are carried out based on the information of their chemical taxonomy. **(B, C)** The principal component analysis (PCA) score plots are shown separately in (B) positive and (C) negative ion modes. **(D, E)** The OPLS-DA score plots are shown in (D) positive and (E) negative ion modes. t[1] represents principal component 1, t[2] represents principal component 2, and the ellipse represents the 95 % confidence interval. **(F, G)** The OPLS-DA permutation test plots are shown in (F) positive and (G) negative ion modes. **(H, I)** The volcano plots for metabolites after ADAMTS9-AS2-Exos treatment are shown in (H) positive and (I) negative ion modes. A9-AS2, ADAMTS9-AS2; Exos, exosomes; OPLS-DA, orthogonal partial least squares discriminant analysis.

Due to the inhibitory role of the ADAMTS9-AS2/Let-7a-5p axis in the Warburg effect, we further investigated whether the ADAMTS9-AS2 exosomes modulated metabolic reprogramming in OSCC cells using UHPLC & tandem mass spectrometry. A total of 926 metabolites were identified after ADAMTS9-AS2-Exos treatment, including lipids and lipid-like molecules, organic acids and derivatives, organic oxygen compounds, and benzenoids (Fig. 6A and Table S1). Based on this metabolite information, an unsupervised principal component analysis model showed a clear trend of separation between the ADAMTS9-AS2-Exos treatment group and the control group in both the cationic and anionic modes, indicating that the two groups were different in terms of metabolite composition (Fig. 6B, C).

To further identify these metabolites and better differentiate between the groups, the orthogonal partial least squares discriminant analysis (OPLS-DA) score plot further showed a clear separation between the ADAMTS9-AS2-Exos group and the control group in both the cationic and anionic modes (Fig. 6D, E). In addition, to avoid overfitting, the OPLS-DA results were evaluated using the OPLS-DA substitution test, with an R2 of 0.9141 in the cationic mode and 0.9881 in the anionic mode (Fig. 6F, G). These results suggest that both cationic and anionic OPLS-DA models in our study are reliable.

Further differential metabolite analysis by volcano plots was constructed (variable importance in project >1; *P* < 0.05) to highlight metabolites within the two groups based on these differential metabolites. In the cationic mode volcano plot, 567 differential ion peaks were detected, with only 39 metabolites identified (Fig. 6H and Table S2). In the anionic mode volcano plot, 359 differential ion peaks were detected, with only 37 metabolites identified (Fig. 6I and Table S3). Moreover, fold change values of metabolite expression differences between the two groups were higher in the cationic mode than in the anionic mode. A total of 76 named metabolites were detected, of which 39 were up-regulated and 37 were down-regulated (Fig. S1).

**Figure 7:**
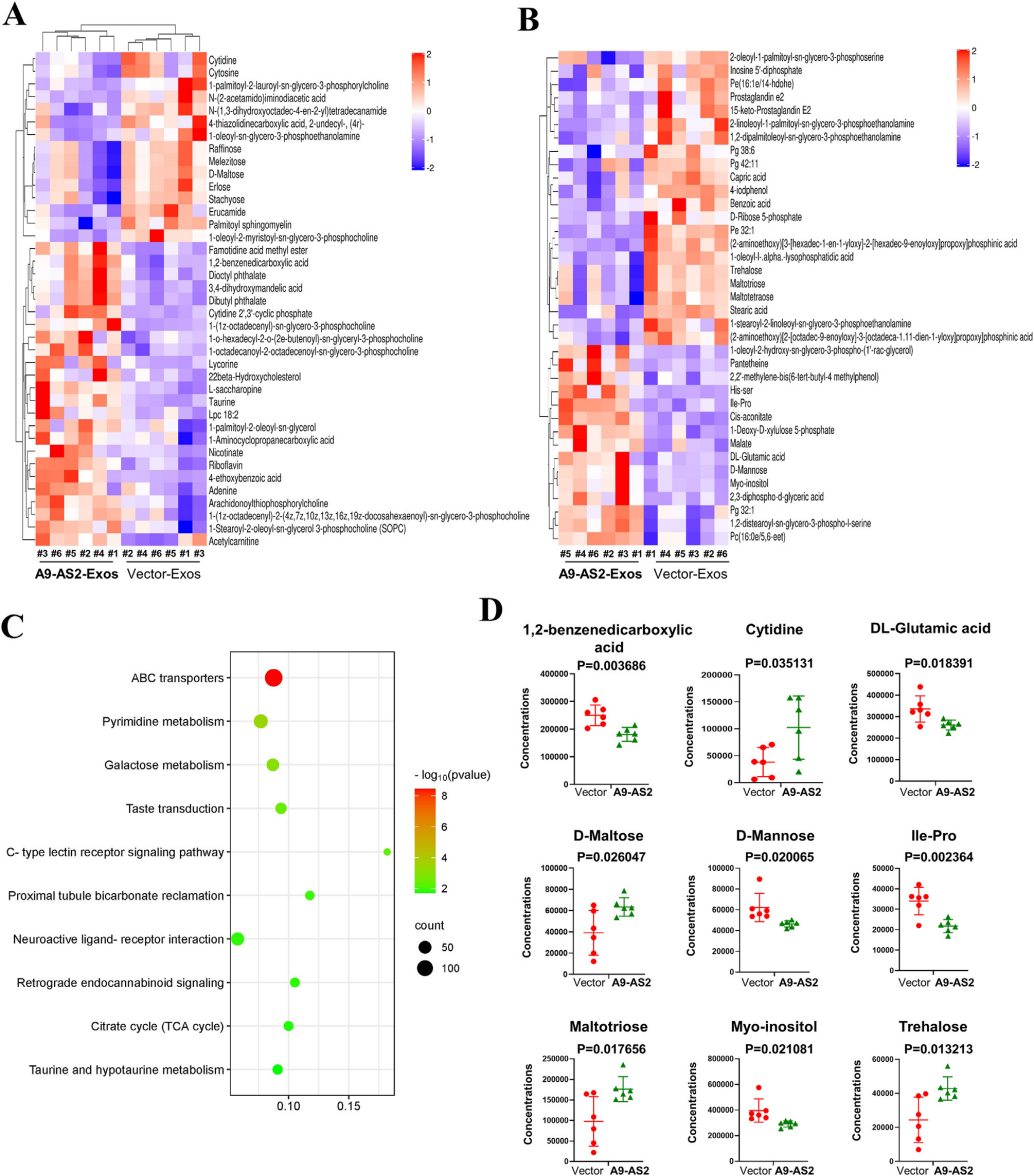
Function analysis of ADAMTS9-AS2-mediated metabolic alterations in oral squamous cell carcinoma cells. **(A, B)** Hierarchical clustering heat map of significantly different metabolites after ADAMTS9-AS2-Exos treatment in (A) positive and (B) negative ion modes. **(C)** Kyoto Encyclopedia of Genes and Genomes (KEGG) pathway enrichment analysis on metabolites after ADAMTS9-AS2-Exos treatment. **(D)** The changes of differential metabolites identified in ABC transporters, pyrimidine metabolism, and galactose metabolism are shown. A9-AS2, ADAMTS9-AS2; Exos, exosomes.

To better understand the significantly different metabolites by ADAMTS9-AS2-Exos, a clustering heat map was constructed (variable importance in project >1; *P* < 0.05), and a clear separation of metabolites was observed between the ADAMTS9-AS2-Exos group and the control group (Fig. 7A, B). These results suggest that ADAMTS9-AS2-Exos modulates metabolic reprogramming in OSCC cells.

### Regulation of metabolic pathways by ADAMTS9-AS2 exosomes in OSCC cells

To further understand the metabolite changes mediated by ADAMTS9-AS2 exosomes, pathway enrichment analysis of differential metabolites was performed. The top 10 enriched pathways were ABC transporters, pyrimidine metabolism, galactose metabolism, taste transduction, C-type lectin receptor signaling pathway, proximal tubule bicarbonate reclamation, neuroactive ligand–receptor interaction, retrograde endocannabinoid signaling, and citrate cycle (TCA cycle) (Fig. 7C and Table S4).

In addition, specific changes in metabolites, including 1,2-benzenedicarboxylic acid, D-mannitic acid and acid, DL-glutamic acid, D-mannose, and Ile–Pro, were significantly increased (*P* < 0.05), whereas cytidine, D-maltose, maltotriose, and trehalose were significantly decreased (*P* < 0.05) in OSCC cells after ADAMTS9-AS2-Exos treatment. Thus, ADAMTS9-AS2-Exos mainly affected ABC transporters in lipid metabolism and pyrimidine metabolism of OSCC cells.

## Discussion

In this study, we show that the ADAMTS9-AS2/let-7a-5p axis suppresses aerobic glycolysis via targeting its downstream effector HK2 and further modulates metabolic reprogramming in OSCC cells. Moreover, HK2 expression is critical for ADAMTS9-AS2/let-7a-5p axis-mediated tumor proliferation of OSCC cells. Furthermore, we found that ADAMTS9-AS2 exosomes significantly regulated metabolic-related pathways, including ABC transporters in lipid metabolism, pyrimidine metabolism, and galactose metabolism through the modulation of multiple metabolites. Thus, we provided a novel regulatory mechanism for how metabolic dysregulation by the ADAMTS9-AS2/let-7a-5p axis in OSCC cells facilitates reduced glycolysis to suppress OSCC progression.

The interplay between lncRNAs and cancer metabolic reprogramming enables tumor cells to adapt to a microenvironment characterized by low oxygen demand and rapid proliferation, contributing to the accelerated cancer proliferation, tumor cell growth, invasion, and metastasis of cancer.^13^^,^^14^ Accumulating evidence has shown that lncRNAs drive cancer metabolic reprogramming by directly or indirectly interacting with key metabolic enzymes and regulatory molecules, further altering related metabolites and disrupting multiple metabolic pathways,^15^ including lncRNAs H19, NBR, and PVT1. For example, lncRNA H19 up-regulates PKM2 expression and activates the LDHA pathway by targeting let-7 and miR-519D-3p,^16^, ^17^, ^18^ further promoting glycolysis and cell growth. Few studies have been conducted to investigate the role and mechanism of lncRNAs in the reprogramming of metabolism in OSCC.

ADAMTS9-AS2 is a functional tumor suppressor that inhibits tumor cell proliferation, invasion, and migration in multiple cancers,^19^ including lung cancer,^20^^,^^21^ esophageal cancer,^22^^,^^23^ gastric cancer,^24^^,^^25^ hepatocellular cancer,^26^ breast cancer,^27^ clear cell renal cell carcinoma,^28^ and bladder cancer,^29^^,^^30^ consistent with our previous findings in OSF and OSCC. Mechanistically, ADAMTS9-AS2 exerts its tumor suppressor functions by targeting the miR-182-5p/FOXF2,^31^ miR-223-3p/TGFBR3,^21^ miR-223-3p/NLRP3,^24^ miR-196b-5p/PPP1R12B,^23^ and miR-27a-3p/FOXO1^28^ axis, or by mediating CDH3 promoter methylation.^22^ ADMATAS9-AS2 can also disrupt PI3K/AKT^12^ and TGF-β^27^ signaling pathways. However, whether metabolic dysregulation is also involved in its tumor suppressive functions is largely unknown.

We found that the metabolic pathway is the most important pathway during the malignant progression of OSF by screening different stages of normal mucosa, OSF, and OSCC tissues. Furthermore, we found that exosome-derived ADAMTS9-AS2 suppressed aerobic glycolysis in OSCC cells and was positively correlated with let-7a-5p expression levels. Let-7a is frequently down-regulated in OSCC tissues and cell lines^32^^,^^33^ and is associated with tumor size and lymph node metastasis, and may serve as a biomarker for poor prognosis in OSCC patients,^33^ which is consistent with our data. Let-7a inhibits OSCC cell proliferation, invasion, and migration by targeting c-Myc and regulating the MAPK/ERK signaling pathway.^32^ In addition, let-7a-5p suppresses chemoresistance in HNSCC by ablating stem-like properties.^34^ Salivary let-7a-5p and miR-3928 could be used as potential diagnostic biomarkers for HNSCC.^33^^,^^35^ These results suggest the importance of let-7a-5p silencing in the development and progression of OSCC.

Our data revealed a potential interaction between ADAMTS9-AS2 and let-7a-5p in OSCC cells. We observed an up-regulation of let-7a-5p in ADAMTS9-AS2-Exos-treated cells. Using siRNA-mediated knockdown of ADAMTS9-AS2, we further confirmed that ADAMTS9-AS2 expression is required for let-7a-5p up-regulation, suggesting positive regulation between ADAMTS9-AS2 and let-7a-5p in OSCC tissues. ADAMTS9-AS2 has also been identified as a driver of neuroblastoma differentiation through the regulation of LIN28B/let-7/MYCN signaling.^36^ Some mechanisms for lncRNA and miRNA interaction have been elucidated, including acting as sponges for miRNAs, competing to bind to the same target mRNA, miRNA generation, and miRNA-mediated lncRNA degradation. However, we did not find any potential binding sites between ADATMS9-AS2 and let-7a-5p for sponges and competition with each other. We thus speculated that the possible regulatory mechanism is that let-7a-5p can be produced from ADAMTS9-AS2 during lncRNA processing by endoribonucleases such as Dicer and Drosha.^37^^,^^38^

We demonstrated a critical role for ADAMTS9-AS2 in metabolic regulation via HK2 in OSCC. We also identified let-7a-5p as a novel inhibitor of HK2, which suppressed the HK2-mediated Warburg effect. These findings suggest that the ADAMTS9-AS2/let-7a-5p/HK2 axis is a promising therapeutic target for OSCC patients. miRNAs repress gene expression by binding to the 3′-UTR of the target mRNA. A number of miRNAs, such as let-7, miR-34a, miR-15b, and miR-143, have been identified to play an important role in modulating metabolism in mammals.^39^^,^^40^ Using bioinformatic prediction and further validation, we have shown that let-7a-5p negatively regulates HK2 by binding to its 3′-UTR. Other downstream targets of let-7a-5p have been reported to regulate metabolism, including HMGA2,^41^ Rictor,^42^ GLUT12,^43^ and PKM2.^42^ Our study has identified an additional mechanism for let-7a-5p in the regulation of glycolysis during OSCC pathogenesis.

Metabolic reprogramming has been defined as a novel therapeutic target for metabolic alterations in tumor cells. Therefore, understanding changes in multiple metabolic pathways in cancer cells may serve as a benchmark for the development of new anti-cancer therapies. Increased glycolysis may promote metabolic reprogramming of normal oral fibroblasts in OSCC, which precedes activation into carcinoma-associated fibroblasts.^44^

In this study, our results demonstrated the role of ADAMTS9-AS2 in the metabolic reprogramming of OSCC cells by regulating the disruption of ABC transporters in lipid and pyrimidine metabolism. ABC transporters are widely recognized as efflux proteins that decrease intracellular concentrations of therapeutic agents, including chemotherapeutic drugs, leading to the development of drug resistance.^45^^,^^46^ Studies have shown that increased expression of ABC transporters may be a driver of drug resistance in OSCC cells, suggesting a role for ADAMTS9-AS2 in drug resistance in OSF and OSCC. In cancer, impaired pyrimidine metabolism not only results in an excess of nucleotides but also affects other metabolic pathways and cellular signaling. The pyrimidine metabolic enzyme dihydroorotate dehydrogenase (DHODH), which is transcriptionally regulated by SOX2, is up-regulated in OSCC tissues.^47^ DHODH inhibition impairs pyrimidine nucleotide synthesis in OSCC, highlighting the key role of pyrimidine metabolism as a potential prognostic and therapeutic target for OSCC treatment.^47^ In addition, increased metabolites by ADAMTS9-AS2 exosomes, such as DL-glutamic acid and D-mannose as anti-cancer agents, facilitate immunotherapy and radiotherapy in cancer.^48^

Future studies are needed to investigate the mechanisms underlying the metabolic changes caused by ADAMTS9-AS2. The roles of metabolites such as DL-glutamic acid and D-mannose, while highlighted as potential anticancer agents, need to be determined for their specific contributions to improve therapeutic efficacy in OSCC. Future *in vivo* OSCC-OSF models will be established to validate the ADAMTS9-AS2/HK2 axis regulating cell proliferation via glycolysis and to evaluate the potential of targeting this axis for therapeutic intervention in OSCC cells.

In conclusion, this is the first study to identify the metabolic function of ADAMTS9-AS2 in OSCC tumorigenesis. The ADAMTS9-AS2/let-7a-5p/HK2 axis plays an important role in the regulation of cancer metabolism and cell proliferation, which has potential implications for cancer therapy. These findings highlight the importance of the ADAMTS9-AS2/let-7a-5p/HK2 axis in controlling the Warburg effect and OSCC progression. Our results provided a global profile of metabolic reprogramming with ADAMTS9-AS2 exosome treatment, which may lead to the development of personalized targeted therapy for the treatment of OSCC.

## CRediT authorship contribution statement

**Shanghui Zhou:** Conceptualization, Data curation, Funding acquisition, Investigation, Methodology, Validation, Writing – original draft, Writing – review & editing. **Jingyu Zhan:** Data curation, Formal analysis, Methodology. **Jia Wang:** Data curation, Formal analysis, Methodology, Validation. **Jingang Yang:** Investigation, Methodology, Resources. **Dahe Zhang:** Data curation, Investigation, Methodology. **Zhenming Li:** Data curation, Formal analysis, Investigation, Methodology. **Yue He:** Resources, Visualization, Writing – review & editing.

## Ethics declaration

This study was approved by the Institutional Review Board of Shanghai Jiaotong University School of Medicine (No. SH9H-2022-TK83-1).

## Data availability

The data are available within the article or its supplementary materials.

## Funding

This project was supported by the 10.13039/501100001809National Natural Science Foundation of China (No. 82460458) and the 10.13039/501100003399Natural Science Foundation of Shanghai, China (No. 21ZR1438200).

## Conflict of interests

The authors declare that they have no known competing financial interests or personal relationships that could have appeared to influence the work reported in this paper.
