# Supplementary material for: lncRNA ADAMTS9-AS2/let-7a-5p axis regulates metabolic reprogramming by targeting HK2 in oral submucous fibrosis-associated oral squamous cell carcinoma
Source: Genes Dis. 2025 May 5;12(6):101670. doi: 10.1016/j.gendis.2025.101670 (PMC12359156; doi:10.1016/j.gendis.2025.101670)
Supplement: Multimedia component 1 [file mmc1.pdf]

Supplementary information

Establishment of stably expressed cell lines

LncRNA ADAMTS9-AS2 lentiviral expression vector was constructed by GeneChem Co. (Shanghai, China). Lentiviral transduction was performed according to the manufacturer's instructions. GFP expression was observed by fluorescence microscopy at approximately 80% fluorescence and 80% cell confluence. To establish stably expressing cell lines, cells were screened with antibiotics at 72 h post-infection.

Exosome isolation and purification

Exosomes were isolated and purified using ExoQuick™ Exosome Precipitation Solution (SBI) according to the manufacturer's protocol. Briefly, cells and cell debris were removed by centrifugation at 3000 × g. Appropriate volume of Exo-Quick Exosome Precipitation Solution (Exo-Quick, SBI, USA) was added to the transferred supernatant and left overnight at +4°C. The Exo-Quick mixture was centrifuged at 1500 × g for 30 min at room temperature. Following centrifugation, the exosome pellets were resuspended in 100μL of 1X PBS for RNA/protein extraction or cell treatment.

Transmission electron microscopy

The exosomes were extracted from the collected cell supernatant using ExoQuick™ Exosome Precipitation Solution (Cat# EXOQ5A-1,EXOQ20A-1, SBI, USA). The exosome pellet was then centrifuged and resuspended in 100 μl PBS. To prepare the sample for imaging, 5-10 μl of the exosome suspension was gently dropped onto the front of a copper grid. After 1 minute, the excess liquid was carefully blotted dry with clean filter paper. Next, 10-20μl of EM solvent was added dropwise, and the excess liquid was again carefully blotted dry with clean filter paper after 1 minute. The sample was then allowed to dry thoroughly before being imaged.

Suppl. Figure 1. Analysis of the fold difference in the expression of significantly different metabolites in (A) positive and (B) negative ion modes, respectively.

Suppl. Table 1. Detailed chemical classification information of each metabolite identified in CAL27 cells after ADAMTS9-AS2 exosome treatment. All metabolites identified by positive and negative ions are classified and counted according to their chemical taxonomy information.

Suppl. Table 2. Significantly different metabolites after ADAMTS9-AS2 exosome treatment in positive ion mode.

Suppl. Table 3. Significantly different metabolites after ADAMTS9-AS2 exosome treatment in negative ion mode.

Suppl. Table 4. KEGG pathway annotation results for the different metabolites regulated by ADAMTS9-AS2 exosomes.

Suppl. fig 1

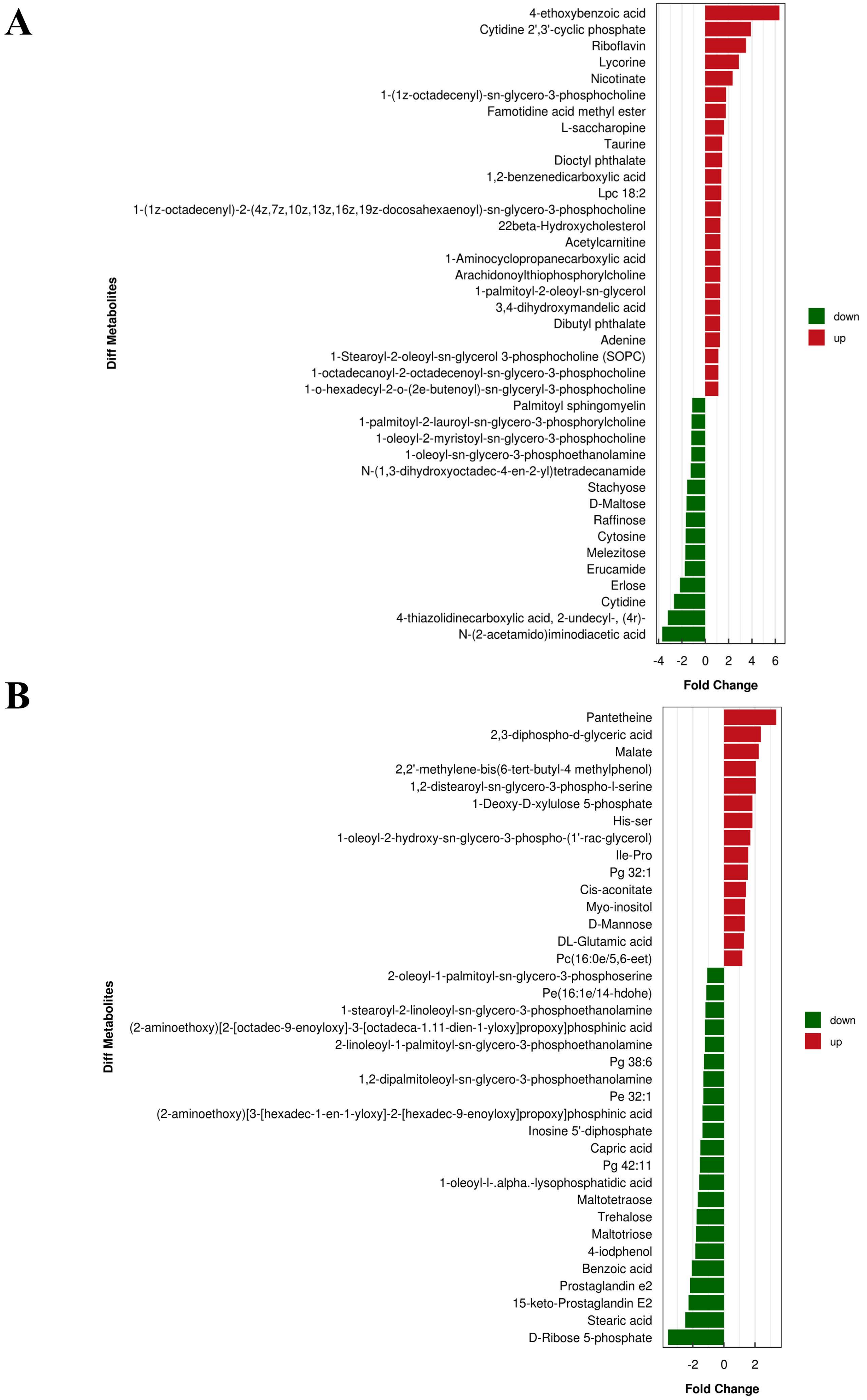
A
4-ethoxybenzoic acid
Cytidine 2',3'-cyclic phosphate
Riboflavin
Lycorine
Nicotinate
1-(1z-octadecenyl)-sn-glycero-3-phosphocholine
Famotidine acid methyl ester
L-saccharopine
Taurine
Dioctyl phthalate
1,2-benzenedicarboxylic acid
Lpc 18:2
1-(1z-octadecenyl)-2-(4z,7z,10z,13z,16z,19z-docosahexaenoyl)-sn-glycero-3-phosphocholine
22beta-Hydroxycholesterol
Acetylcarnitine
1-Aminocyclopropanecarboxylic acid
Arachidonoylthiophosphorylcholine
1-palmitoyl-2-oleoyl-sn-glycerol
3,4-dihydroxymandelic acid
Dibutyl phthalate
Adenine
1-Stearoyl-2-oleoyl-sn-glycerol 3-phosphocholine (SOPC)
1-octadecanoyl-2-octadecenoyl-sn-glycero-3-phosphocholine
1-o-hexadecyl-2-o-(2e-butenoyl)-sn-glyceryl-3-phosphocholine
Palmitoyl sphingomyelin
1-palmitoyl-2-lauroyl-sn-glycero-3-phosphorylcholine
1-oleoyl-2-myristoyl-sn-glycero-3-phosphocholine
1-oleoyl-sn-glycero-3-phosphoethanolamine
N-(1,3-dihydroxyoctadec-4-en-2-yl)tetradecanamide
Stachyose
D-Maltose
Raffinose
Cytosine
Melezitose
Erucamide
Erlose
Cytidine
4-thiazolidinecarboxylic acid, 2-undecyl-, (4r)-
N-(2-acetamido)iminodiacetic acid
Diff Metabolites
-4 -2 0 2 4 6
Fold Change
down
up
B
Pantetheine
2,3-diphospho-d-glyceric acid
Malate
2,2'-methylene-bis(6-tert-butyl-4 methylphenol)
1,2-distearoyl-sn-glycero-3-phospho-l-serine
1-Deoxy-D-xylulose 5-phosphate
His-ser
1-oleoyl-2-hydroxy-sn-glycero-3-phospho-(1'-rac-glycerol)
Ile-Pro
Pg 32:1
Cis-aconitate
Myo-inositol
D-Mannose
DL-Glutamic acid
Pc(16:0e/5,6-eet)
2-oleoyl-1-palmitoyl-sn-glycero-3-phosphoserine
Pe(16:1e/14-hdohe)
1-stearoyl-2-linoleoyl-sn-glycero-3-phosphoethanolamine
(2-aminoethoxy)[2-[octadec-9-enoyloxy]-3-[octadeca-1.11-dien-1-yloxy]propoxy]phosphinic acid
2-linoleoyl-1-palmitoyl-sn-glycero-3-phosphoethanolamine
Pg 38:6
1,2-dipalmitoleoyl-sn-glycero-3-phosphoethanolamine
Pe 32:1
(2-aminoethoxy)[3-[hexadec-1-en-1-yloxy]-2-[hexadec-9-enoyloxy]propoxy]phosphinic acid
Inosine 5'-diphosphate
Capric acid
Pg 42:11
1-oleoyl-l-.alpha.-lysophosphatidic acid
Maltotetraose
Trehalose
Maltotriose
4-iodphenol
Benzoic acid
Prostaglandin e2
15-keto-Prostaglandin E2
Stearic acid
D-Ribose 5-phosphate
Diff Metabolites
-2 0 2
Fold Change
down
up
